# α2-3-Sialylated Glycoproteins Attenuate *Streptococcus mutans* Virulence and Associated Host Inflammatory Responses

**DOI:** 10.3390/ijms27156562

**Published:** 2026-07-23

**Authors:** Xiameng Ren, Lingyun Wei, Tao Liu, Min Wang, Ziyi Chang, Jian Shu, Zheng Li

**Affiliations:** 1 Laboratory for Functional Glycomics, Faculty of Life Science & Medicine, Northwest University, Xi’an 710069, China; 18591800666@163.com (X.R.); wly_18693074754@163.com (L.W.); w1106828920@163.com (M.W.); changziyi15211@163.com (Z.C.); 2 School of Medicine, Faculty of Life Science & Medicine, Northwest University, Xi’an 710069, China; 19829767605@163.com

**Keywords:** SAα2-3Gal glycans, *Streptococcus mutans*, host-microbe interactions, salivary glycosylation, oral microbiome

## Abstract

Glycans attached to host glycoproteins play an important role in regulating oral microbial colonization and maintaining community balance. Our previous studies revealed that children exhibit lower levels of α2-3 sialylated glycan structures (SAα2-3Gal) than adults, raising the possibility that this age-associated glycan deficiency contributes to the heightened cariogenicity of *Streptococcus mutans*. In this study, sialylated glycoproteins (Sia-GP) and corresponding desialylated controls (DeSia-GP and α2,3-DeSia-GP) were prepared from bovine milk-derived glycoproteins to investigate the functional contribution of SAα2-3Gal. Their effects on *S. mutans* were evaluated by assessing bacterial growth, acid production, biofilm formation and extracellular polysaccharide synthesis, while a human oral keratinocytes (HOK cells) infection model was used to examine epithelial cell viability, wound healing, and infection-associated inflammatory responses. The results showed that Sia-GP exerted only a transient inhibitory effect on early bacterial growth without affecting final biomass, but significantly attenuated acidogenic activity, biofilm formation, and extracellular polysaccharides production in a concentration-dependent manner. These inhibitory effects were markedly attenuated following removal of α2-3-linked sialic acids, demonstrating the essential role of SAα2-3Gal. In addition, Sia-GP alleviated *S. mutans*-induced cellular damage in HOK cells by improving cell viability, colony formation, and migration, while suppressing infection-associated inflammatory signaling. Collectively, these findings demonstrate that SAα2-3Gal effectively limits multiple virulence traits of *S. mutans* and attenuates *S. mutans*-induced damage in oral epithelial cells. This study provides mechanistic insights into glycan-mediated regulation of host–microbe interactions and suggests that the naturally low abundance of SAα2-3Gal in children may increase their susceptibility to *S. mutans* infection and caries development.

## 1. Introduction

The balance between the host and its resident microbiota is influenced by multiple factors, including immune responses, environmental conditions, and host molecular signals [1,2]. Among these, glycans on host glycoprotein are critical for mediating microbial adhesion, community composition, and host–microbe interactions [3,4]. These glycans regulate microbial behavior through multiple mechanisms. Some serve as selective nutrient sources that support the growth of specific bacterial populations, and thereby influencing community dynamics, whereas others act as molecular decoys or signaling modulators that interfere with microbial adhesion or communication, reducing virulence and altering host–microbe interactions [5,6]. Such functional diversity is largely attributable to the remarkable structural complexity of glycans, which enables them to participate in a wide range of molecular interactions as a central axis of interspecies crosstalk, thereby dictating microbial colonization while shaping host immune surveillance and community stability [5,7,8].

In the oral cavity, host-derived glycoconjugates serve as a key class of mediators of microbial homeostasis. Alterations in host glycosylation are increasingly recognized to shape the assembly and functional behavior of the oral microbiome [9,10]. Variations in specific glycan motifs have been shown to regulate microbial adhesion, colonization efficiency, and interspecies interactions, ultimately contributing to dysbiosis [11,12,13]. Salivary glycoproteins represent major carriers of these glycan structures in the oral environment, and their glycosylation patterns provide a functional basis for host–microbe interactions [14,15]. For instance, O-glycans derived from salivary MUC5B have been shown to suppress quorum sensing and attenuate virulence in *Streptococcus mutans* (*S. mutans*), including downregulation of competence signaling pathways and inhibition of genetic transformation [16]. Consistently, a cohort study further demonstrated that salivary protein glycopatterns naturally regulate the oral microbiome in vivo, as reduced salivary fucosylation in gastric cancer patients was associated with increased *Aggregatibacter segnis* abundance via enhanced adhesion and immune evasion [17]. These findings collectively highlight that host glycosylation is not merely a passive structural feature of the oral environment but an active regulator of microbial ecology.

Notably, our previous study has shown that α2-3 sialylated glycans (SAα2-3Gal) were reduced in the oral cavity of children compared with adults [18]. As terminal motifs, SAα2-3Gal participate in host–microbe interactions by serving as recognition elements for bacterial surface components or by modulating host responses, thereby influencing the colonization and activity of oral microbiota [19]. *S. mutans*, a primary cariogenic pathogen known to interact with host glycan structures during colonization, is among the most prevalent bacteria in the pediatric oral microbiome [20,21]. Given the high prevalence of dental caries in children [22], and that alterations in specific glycans can profoundly reshape microbial adhesion and colonization, it remains unclear whether the reduction in SAα2-3Gal is associated with enhanced *S. mutans* colonization and virulence in the pediatric oral cavity.

In this study, the role of SAα2-3Gal in modulating *S. mutans* virulence was investigated using intact sialylated glycoproteins (Sia-GP) and their selectively desialylated controls. This approach enabled the assessment of how terminal sialic acid residues influence bacterial behavior and host cell responses, thereby providing insight into glycan-mediated regulation of microbial pathogenicity. This aligns with emerging anti-virulence strategies that target pathogenicity mechanisms rather than bacterial viability, offering a promising approach to mitigate dysbiosis while minimizing selective pressure for resistance [23,24], and informing potential glycan-based strategies for maintaining microbial homeostasis.

## 2. Results

### 2.1. Effect of SAα2-3Gal Glycan Structures on the Growth of S. mutans

**Figure 1:**
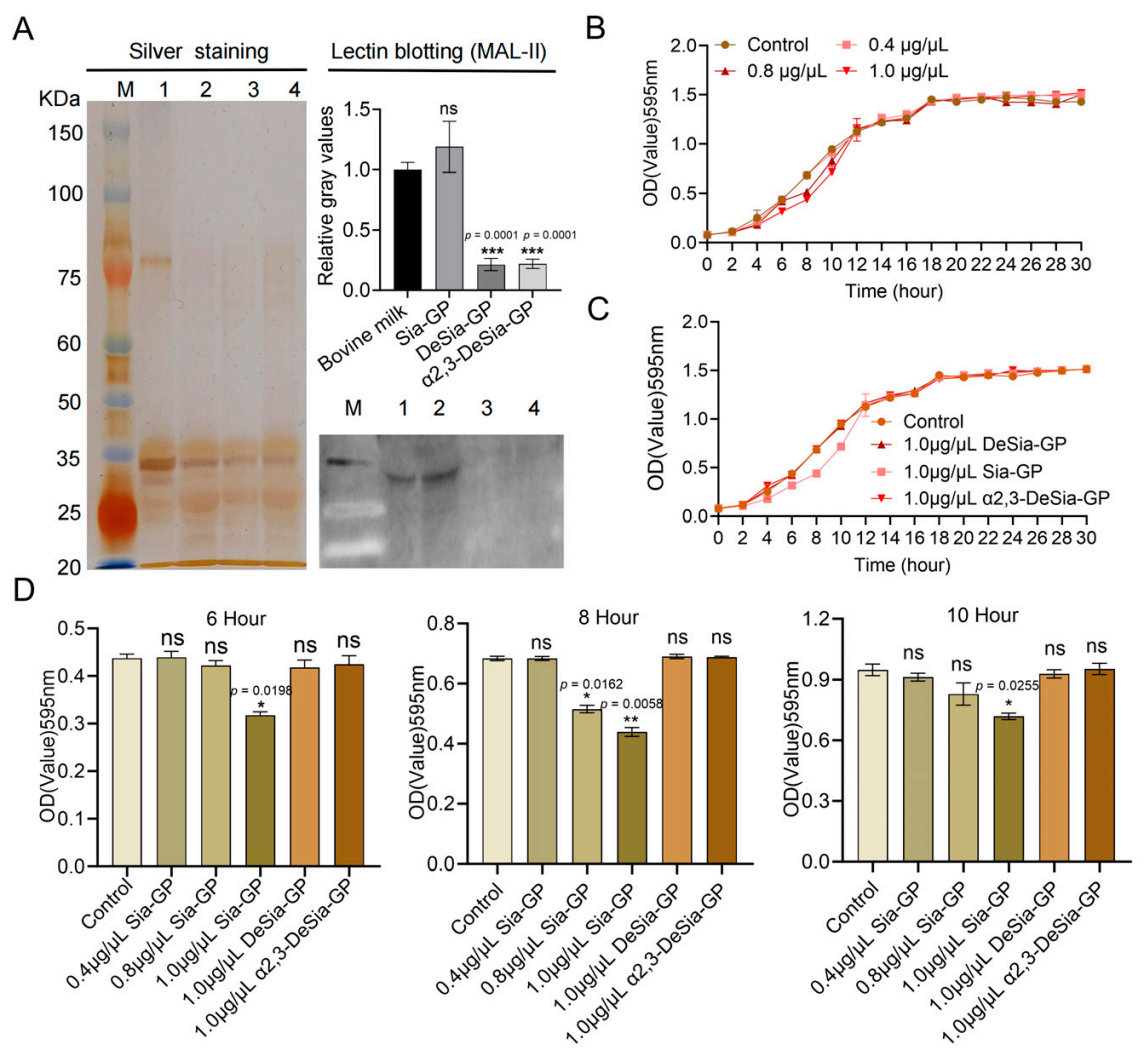
Characterization of glycoproteins and their effects on *S. mutans* growth. (**A**) SDS-PAGE followed by silver staining, lectin blotting using biotinylated MAL-II, and corresponding gray value analysis of bovine milk (lane 1), Sia-GP (lane 2), DeSia-GP (lane 3), and α2,3-DeSia-GP (lane 4). (**B**) Growth curves of *S. mutans* cultured with different concentrations of Sia-GP (0, 0.4, 0.8, and 1.0 μg/μL). (**C**) Growth curves of *S. mutans* treated with Sia-GP, DeSia-GP and α2,3-DeSia-GP. (**D**) Comparison of *S. mutans* growth at 6, 8, and 10 h under Sia-GP treatment at different concentrations (0, 0.4, 0.8, and 1.0 μg/μL), together with DeSia-GP and α2,3-deSia-GP treatments at 1.0 μg/μL. Sia-GP—sialylated glycoprotein; DeSia-GP—desialylated glycoprotein; α2,3-DeSia-GP—α2,3-desialylated glycoprotein. Data are presented as mean ± SD from three independent experiments (*n* = 3), each performed in triplicate. ns—not significant; * *p* < 0.05, ** *p* < 0.01, *** *p* < 0.001.

The preparation and desialylation efficiency of glycoproteins were first evaluated. SDS-PAGE followed by silver staining showed comparable band patterns among milk, Sia-GP, DeSia-GP, and α2,3-DeSia-GP, with major bands located around 35 kDa, indicating that desialylation did not induce detectable protein degradation or aggregation and that overall protein integrity was preserved (Figure 1A). Lectin blotting using biotinylated MAL-II, together with gray value analysis, demonstrated a marked reduction in α2-3-linked sialic acid signals in both the α2,3-DeSia-GP and DeSia-GP groups after enzymatic treatment (Figure 1A). The effect of SAα2-3Gal glycan structures on the growth of *S. mutans* was assessed by measuring optical density (OD_595_) over time. Across the Sia-GP concentration gradient, all groups exhibited similar OD595 values during the initial 0–4 h. During the early exponential phase (6–10 h), growth in the Sia-GP-treated groups, particularly at higher concentrations, was slightly delayed compared to the control group. However, after approximately 10 h, the growth curves began to converge, and no significant differences were observed thereafter (Figure 1B). A comparison of different glycoproteins revealed that, compared with the DeSia-GP and α2,3-DeSia-GP groups, bacterial growth in the Sia-GP-treated group was slightly delayed during the early growth phase, while the growth patterns of the other two groups were similar to the control group (Figure 1C). Statistical analysis confirmed that most differences were not significant, with only minor variations at individual early time points (Figure 1D). Overall, Sia-GP exerted only a minor and transient effect on the growth of *S. mutans*, without affecting its final biomass.

### 2.2. Effect of SAα2-3Gal Glycan Structures on the Acidogenic Activity and Acid Tolerance of S. mutans

**Figure 2:**
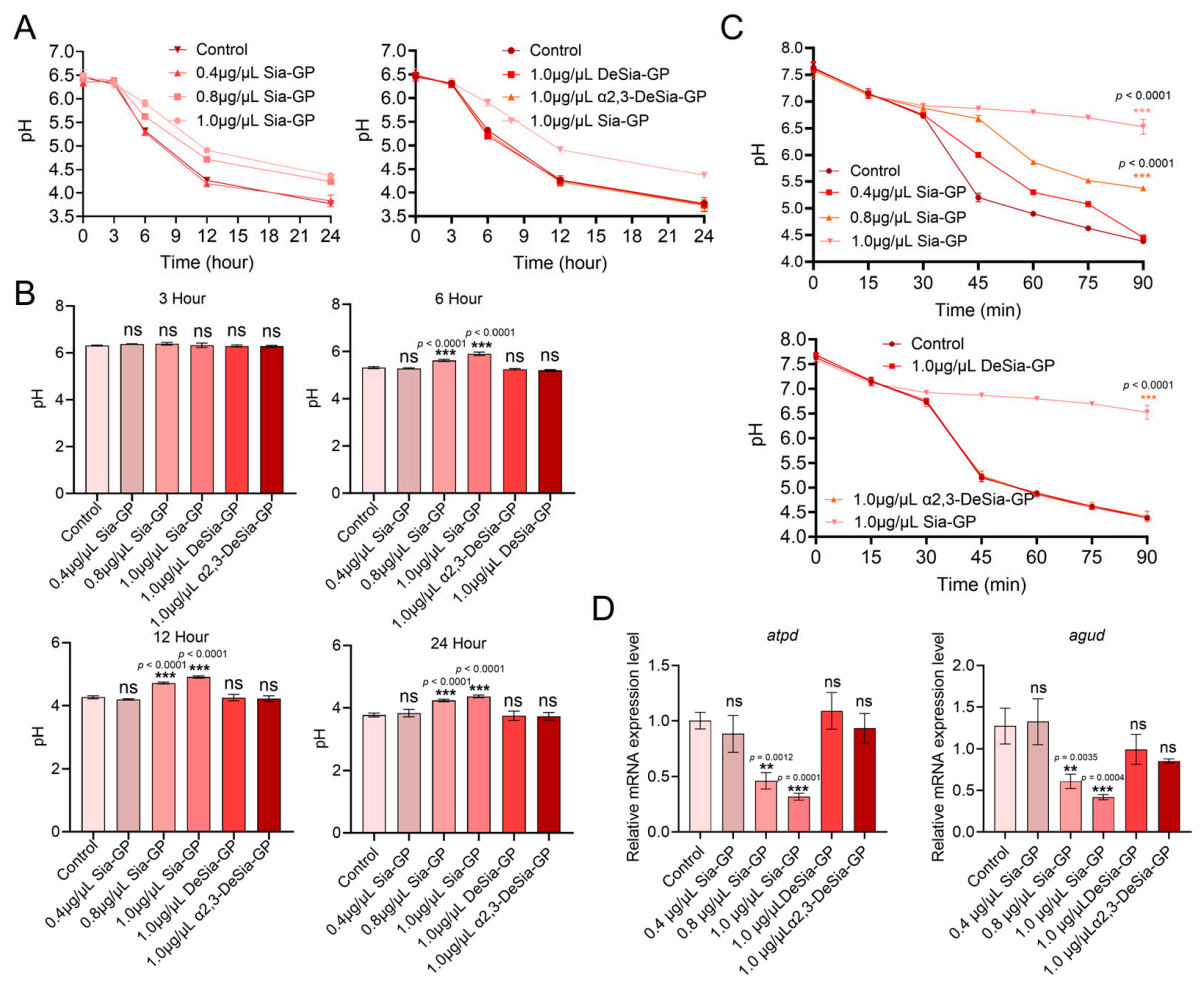
Acid production and acid tolerance of *S. mutans* under different glycoprotein treatments. (**A**) Changes in pH values of *S. mutans* cultures over time under Sia-GP treatment at different concentrations (0, 0.4, 0.8, and 1.0 μg/μL) and under desialylated DeSia-GP and α2,3-deSia-GP treatments compared with the control group. (**B**) Comparison of pH values at 3, 6, 12, and 24 h under different Sia-GP concentrations (0, 0.4, 0.8, and 1.0 μg/μL) and under desialylated DeSia-GP, and α2,3-deSia-GP treatments. (**C**) Glycolytic activity of *S. mutans* assessed by a glucose-stimulated pH drop assay under different glycoprotein treatments. (**D**) Relative mRNA expression levels of acidogenesis- and acid tolerance-related genes *atpD* and *aguD* in *S. mutans* treated with different glycoproteins for 24 h. Sia-GP—sialylated glycoprotein; DeSia-GP—desialylated glycoprotein; α2,3-DeSia-GP—α2,3-desialylated glycoprotein. Data are presented as mean ± SD from three independent experiments (*n* = 3), each performed in triplicate. ns, not significant; ** *p* < 0.01, *** *p* < 0.001.

As acidogenesis represents a primary virulence mechanism of *S. mutans*, the acidogenic potential under different glycoprotein treatments was evaluated by measuring the pH of culture supernatants at 0, 3, 6, 12, and 24 h. In the Sia-GP concentration gradient (0–1.0 μg/μL), all groups started at a similar pH and gradually decreased over time. When different glycoproteins were compared, Sia-GP showed a clear inhibitory effect on acid production in a concentration-dependent manner, with significant differences observed at 0.8 and 1.0 μg/μL, whereas DeSia-GP and α2,3-DeSia-GP exhibited effects comparable to the control at 1.0 μg/μL (Figure 2A,B). Consistent with this observation, glucose-stimulated bacterial suspensions exhibited a reduced rate of acidification in the Sia-GP group, indicating impaired glycolytic acid production (Figure 2C).

To further explore the molecular basis of the observed reduction in acidification, qPCR analysis was performed on the acidogenesis- and acid tolerance-related genes *atpD* and *aguD*. As shown in Figure 2D, treatment with Sia-GP (0.8 and 1.0 μg/μL) significantly downregulated the mRNA expression levels of both *atpD* and *aguD* compared with the control group. In contrast, the desialylated forms (DeSia-GP and α2,3-DeSia-GP) at 1.0 μg/μL showed no significant changes in the expression of these genes. These findings suggested that Sia-GP reduced the requirement for acid-adaptive responses in *S. mutans*, consistent with an overall attenuation of acidogenic activity, and that SAα2-3Gal structures contributed to this effect.

### 2.3. Effect of SAα2-3Gal Glycan Structures on S. mutans Biofilm Formation

**Figure 3:**
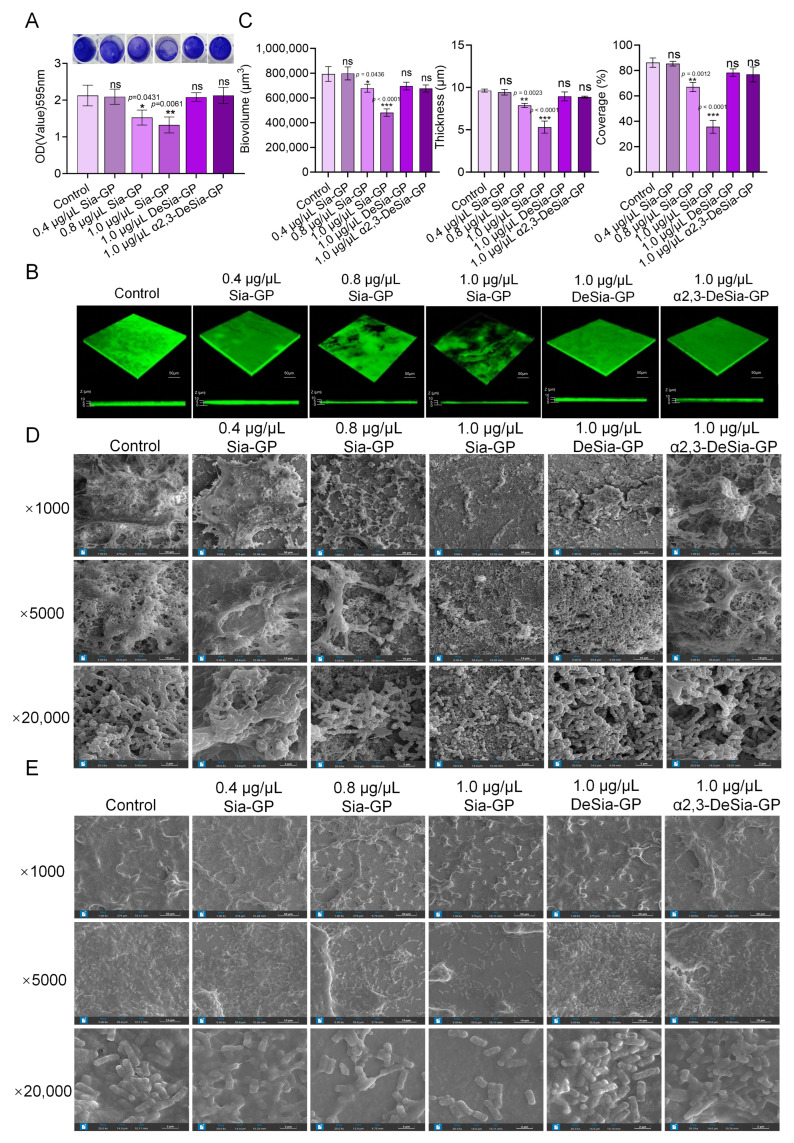
Biofilm formation of *S. mutans* under different glycoprotein treatments. (**A**) Quantification of *S. mutans* biofilm biomass by crystal violet staining assay. Sia-GP was tested at different concentrations (0, 0.4, 0.8, and 1.0 μg/μL), together with DeSia-GP and α2,3-DeSia-GP at 1.0 μg/μL. (**B**) Quantitative analysis of biofilm biomass, thickness, and surface coverage determined by confocal laser scanning microscopy (CLSM). (**C**) Representative three-dimensional CLSM images of *S. mutans* biofilms under different glycoprotein treatments. (**D**) Representative SEM images of *S. mutans* biofilms formed on saliva-coated hydroxyapatite (HA) discs under different treatment conditions. (**E**) Representative SEM images of *S. mutans* biofilms formed on conventional abiotic surfaces under different treatment conditions. Sia-GP—sialylated glycoprotein; DeSia-GP—desialylated glycoprotein; α2,3-DeSia-GP—α2,3-desialylated glycoprotein. Data are presented as mean ± SD from three independent experiments (*n* = 3), each performed in triplicate. ns—not significant; * *p* < 0.05, ** *p* < 0.01, *** *p* < 0.001.

As biofilm formation is a key virulence factor of *S. mutans*, biofilm biomass was first quantified by crystal violet staining after treatment with different glycoproteins. As shown in Figure 3A, Sia-GP decreased OD595 values in a concentration-dependent manner. No significant effect was observed at 0.4 μg/μL, whereas 0.8 and 1.0 μg/μL Sia-GP significantly reduced biofilm formation compared with the control group. To further characterize the effects of Sia-GP on biofilm architecture, three-dimensional imaging analysis was performed. Compared with the control group, Sia-GP-treated biofilms exhibited reduced bacterial accumulation and a less developed biofilm structure. Quantitative analysis showed that Sia-GP significantly decreased biofilm biomass, biofilm thickness and surface coverage, whereas DeSia-GP and α2,3-DeSia-GP showed no significant differences compared with the control group (Figure 3B,C). The effects of Sia-GP on biofilm formation were subsequently examined on hydroxyapatite (HA) discs to mimic an enamel-like surface. As shown in Figure 3C, Sia-GP treatment resulted in reduced bacterial colonization and a less dense biofilm structure on HA surfaces, whereas dense and organized biofilms were observed in the DeSia-GP and α2,3-DeSia-GP groups. Similar morphological differences were also observed on conventional abiotic surfaces (Figure 3D,E). Collectively, these findings indicate that SAα2-3Gal structures reduce biofilm biomass and impair biofilm architecture in *S. mutans*.

### 2.4. Effect of SAα2-3Gal Glycan Structures on Extracellular Polysaccharide (EPS) Production and Glucosyltransferase Expression in S. mutans

**Figure 4:**
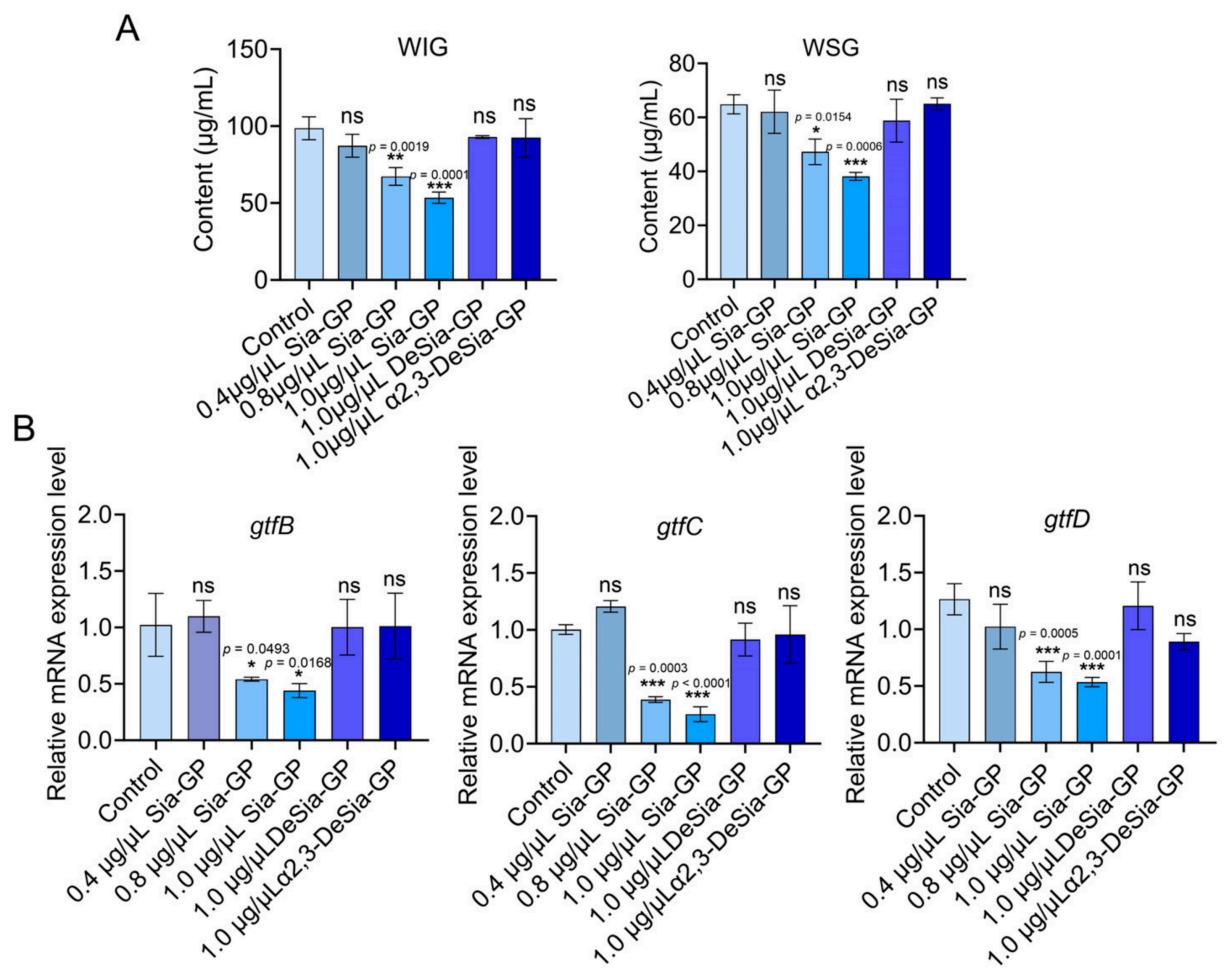
EPS production and glucosyltransferase gene expression in *S. mutans* under different glycoprotein treatments. (**A**) Quantification of WIG and WSG produced by *S. mutans* after 24 h of incubation. (**B**) Relative expression levels of glucosyltransferase genes (*gtfB*, *gtfC*, and *gtfD*) in *S. mutans*, determined by quantitative real-time PCR. WIG—water-insoluble glucan; WSG—water-soluble glucan; Sia-GP—sialylated glycoprotein; DeSia-GP—desialylated glycoprotein; α2,3-DeSia-GP—α2,3-desialylated glycoprotein. Data are presented as mean ± SD from three independent experiments (*n* = 3), each performed in triplicate. ns, not significant; * *p* < 0.05, ** *p* < 0.01, *** *p* < 0.001.

Since extracellular polysaccharides (EPS) synthesized by glucosyltransferases constitute the primary structural scaffold of *S. mutans* biofilms, the effects of SAα2-3Gal glycan structures on EPS production and the transcriptional levels of glucosyltransferase gene (*gtfB*, *gtfC*, *gtfD*) were evaluated after 24 h incubation. Sia-GP at 0.8 μg/μL and 1.0 μg/μL significantly reduced both water-insoluble glucan (WIG) and water-soluble glucan (WSG) production compared with the control group, whereas DeSia-GP and α2,3-DeSia-GP had no effect (Figure 4A). Quantitative analysis of WIG and WSG concentrations was summarized in Table A1. Consistently, qPCR analysis showed that 0.8 μg/μL and 1.0 μg/μL Sia-GP significantly downregulated the transcription of *gtfB*, *gtfC*, and *gtfD* compared with the control group. In contrast, DeSia-GP and α2,3-DeSia-GP did not significantly affect the expression of these genes (Figure 4B). This suggested that the inhibitory effect of Sia-GP on glucosyltransferase gene expression was associated with the presence of SAα2-3Gal structures.

### 2.5. Molecular Docking and Molecular Dynamics Simulation of SAα2-3Gal Glycan Structures with S. mutans Glucosyltransferases

**Figure 5:**
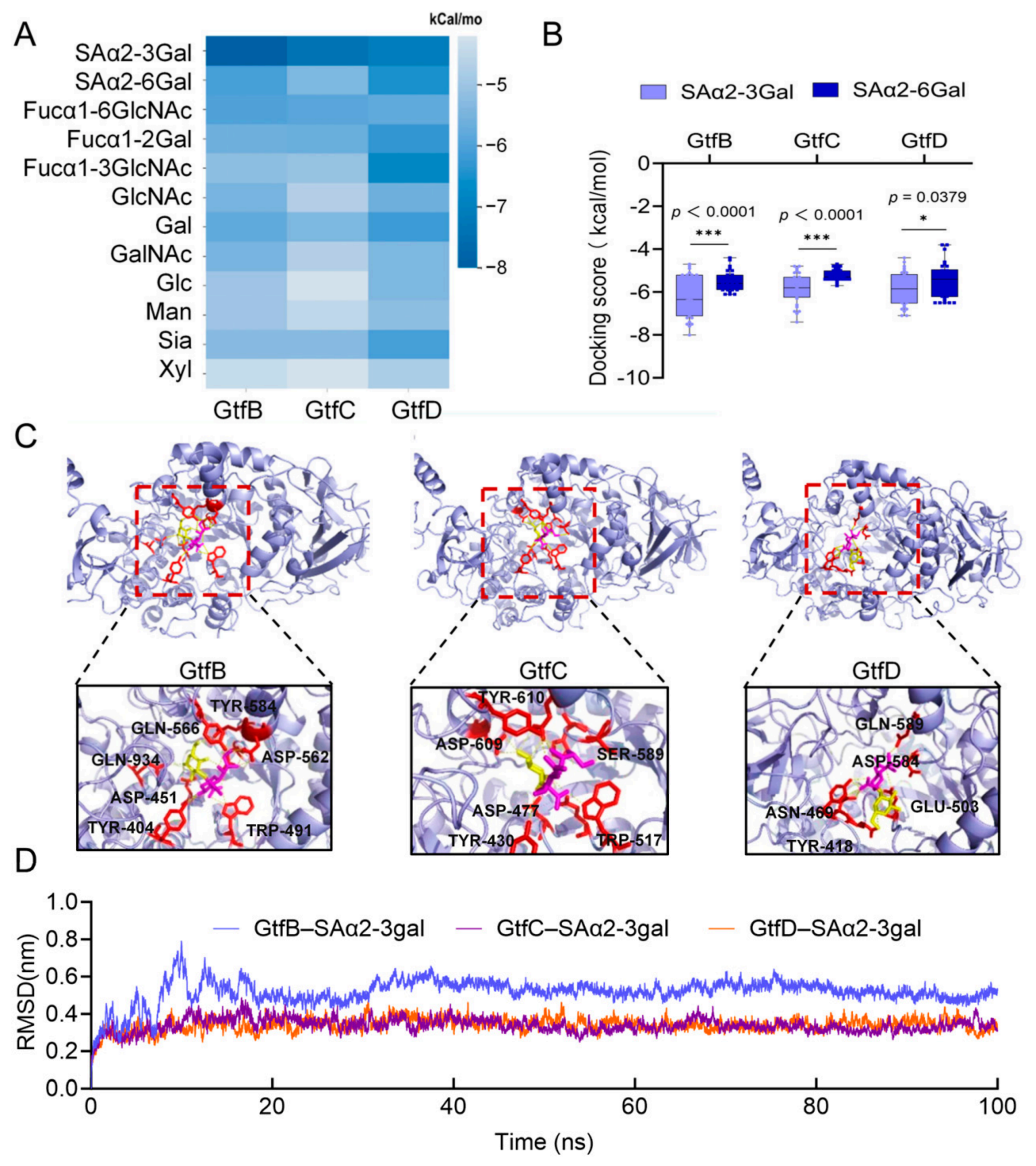
Molecular docking and molecular dynamics simulation of SAα2-3Gal with *S. mutans* glucosyltransferases. (**A**) Heatmap showing the docking binding energies of different glycan structures with GtfB, GtfC, and GtfD. More negative docking scores indicate stronger predicted binding affinities. (**B**) Binding energy distributions obtained from 30 independent molecular docking runs of SAα2-3Gal and SAα2-6Gal against GtfB, GtfC, and GtfD. Statistical significance was evaluated using the Wilcoxon matched-pairs signed-rank test. (**C**) Representative docking models showing the predicted interactions between SAα2-3Gal and GtfB, GtfC, and GtfD. Galactose (Gal) residues are shown as yellow sticks, sialic acid (SA) residues are shown as purple sticks, interacting amino acid residues are shown as red sticks, and hydrogen bonds are indicated by yellow dashed lines. The enlarged binding regions are highlighted by red dashed boxes. (**D**) RMSD trajectories of the SAα2-3Gal–GtfB, SAα2-3Gal–GtfC, and SAα2-3Gal–GtfD complexes during 100 ns molecular dynamics simulations. The corresponding MM/GBSA binding free energies are summarized in Table A3. * *p* < 0.05, *** *p* < 0.001.

Molecular docking simulations were performed to computationally explore the potential interactions between SAα2-3Gal and glucosyltransferases of *S. mutans*. Cluster analysis showed that SAα2-3Gal adopted stable predicted binding poses with GtfB, GtfC, and GtfD. The docking energies indicated that SAα2-3Gal exhibited the strongest binding among all tested glycans, with −8.0, −7.4, and −7.1 kcal/mol for GtfB, GtfC, and GtfD, respectively, whereas other carbohydrates displayed weaker interactions (Figure 5A and Table A2). To further evaluate the reproducibility and structural preference of the predicted interactions, thirty independent docking runs were performed for SAα2-3Gal and the structurally related reference glycan SAα2-6Gal against GtfB, GtfC, and GtfD. The repeated docking results showed consistent binding energy distributions, with SAα2-3Gal exhibiting more favorable predicted docking scores than SAα2-6Gal across all three glucosyltransferases (Figure 5B), suggesting a potential preference of *S. mutans* glucosyltransferases for SAα2-3Gal glycan structures in the computational models. Several interacting residues were located within or adjacent to structurally important regions of these enzymes. Although the predicted binding orientations varied slightly among the three glucosyltransferases, several interacting residues were located within or adjacent to the conserved catalytic region of the enzymes. Multiple hydrogen-bonding and polar contacts were identified in the docking models, supporting the structural plausibility of the predicted glycan–protein association (Figure 5C).

To further evaluate the stability of the predicted complexes, 100 ns molecular dynamics simulations were performed for the SAα2-3Gal–GtfB, SAα2-3Gal–GtfC, and SAα2-3Gal–GtfD complexes. The RMSD trajectories showed that all three complexes gradually reached equilibrium after approximately 40 ns and remained stable for the rest of the simulation (Figure 5D). The average RMSD values of the GtfB, GtfC, and GtfD complexes were 0.52, 0.31, and 0.32 nm, respectively, indicating that SAα2-3Gal binding did not induce substantial conformational changes in the glucosyltransferases. MM/GBSA calculations further revealed favorable binding free energies for the SAα2-3Gal–GtfB, SAα2-3Gal–GtfC, and SAα2-3Gal–GtfD complexes, with ΔGbind values of −30.38 ± 2.25, −23.03 ± 1.77, and −24.41 ± 5.71 kcal/mol, respectively (Table A4). These results further supported the stability of the predicted glycan–protein complexes. Collectively, molecular docking and molecular dynamics simulations supported the structural stability of the predicted SAα2-3Gal–Gtfs complexes. These findings suggest that these glucosyltransferases may represent computationally predicted candidate proteins potentially involved in SAα2-3Gal-associated interactions.

### 2.6. Effects of SAα2-3Gal Glycan Structures on S. mutans-Induced Epithelial Cell Injury

As oral epithelial cells serve as the first line of defense against bacterial invasion, the effects of SAα2-3Gal glycan structures on *S. mutans*-induced alterations in epithelial cell viability, proliferative capacity, and migratory ability were evaluated.

#### 2.6.1. Effects of Sia-GP on HOK Cell Viability

**Figure 6:**
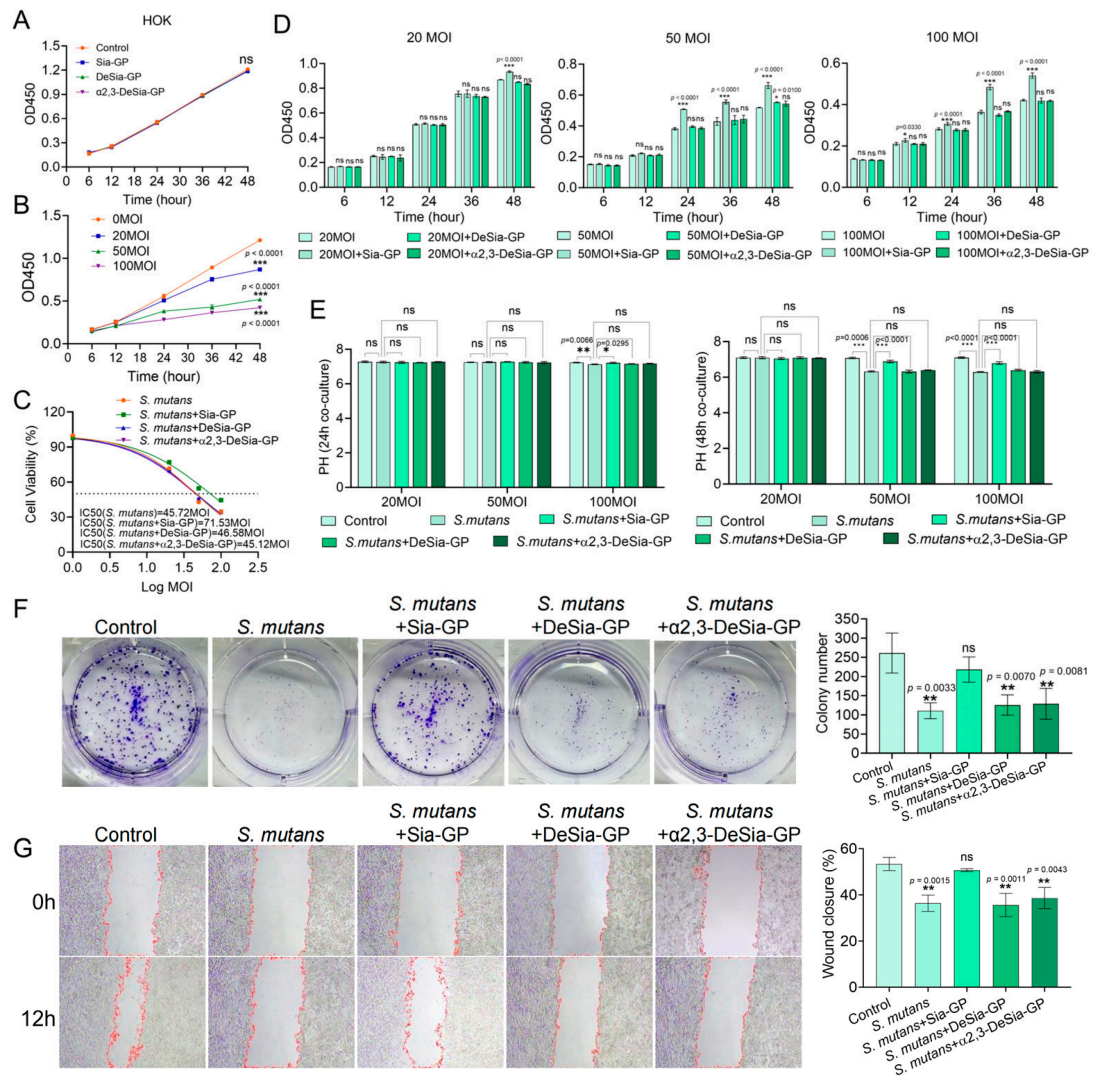
Effects of SAα2-3Gal glycan structures on HOK cell viability, clonogenicity, and migration under *S. mutans* infection. (**A**) Cell viability of HOK cells treated with Sia-GP, DeSia-GP, or α2,3-DeSia-GP alone at 1 μg/μL for 48 h. (**B**) Cell viability of HOK cells after infection with *S. mutans* at MOI 0, 20, 50, and 100. (**C**) IC50 values of *S. mutans* toward HOK cells under different glycoprotein treatments. (**D**) Comparison of the effects of Sia-GP, DeSia-GP, and α2,3-DeSia-GP against *S. mutans*-induced cytotoxicity. (**E**) pH values of culture supernatants measured after 24 h and 48 h of *S. mutans* infection under different treatment conditions. (**F**) Representative images of colony-formation assays showing the clonogenic capacity of HOK cells. Sia-GP partially restored colony formation impaired by *S. mutans* infection, whereas DeSia-GP and α2,3-DeSia-GP exhibited weaker effects. (**G**) Representative wound-healing images showing cell migration. Sia-GP partially alleviated the impairment of wound closure induced by *S. mutans* infection, while DeSia-GP and α2,3-DeSia-GP groups showed diminished recovery. Sia-GP—sialylated glycoprotein; DeSia-GP—desialylated glycoprotein; α2,3-DeSia-GP—α2,3-desialylated glycoprotein. Data are presented as mean ± SD from three independent experiments (*n* = 3), each performed in triplicate. ns—not significant; * *p* < 0.05, ** *p* < 0.01, *** *p* < 0.001.

CCK-8 assay was first used to evaluate the effects of Sia-GP and its deglycosylated forms on HOK cells. As shown in Figure 6A, Sia-GP, DeSia-GP, and α2,3-DeSia-GP alone showed no obvious cytotoxicity at 1 μg/μL. Subsequently, *S. mutans* infection induced a significant reduction in HOK cell viability in a multiplicity of infection (MOI)-dependent manner, with progressively decreased viability observed at MOI 20, 50, and 100 (Figure 6B). Under infection conditions, treatment with Sia-GP, DeSia-GP, and α2,3-DeSia-GP resulted in differential effects on *S. mutans*-induced reductions in HOK cell viability. Compared with the untreated infection group, Sia-GP treated groups showed higher cell viability across conditions, whereas DeSia-GP and α2,3-DeSia-GP showed cell viability levels closer to the infection group. Consistently, IC50 analysis showed that Sia-GP treatment increased the IC50 value under *S. mutans* infection conditions compared with the untreated infection group (Figure 6C,D).

To assess infection-associated acidification, the pH of culture supernatants was measured after 24 h and 48 h of *S. mutans* infection (Figure 6E). No significant differences in pH were observed among groups at MOIs of 20 and 50 after 24 h, whereas a significant decrease was detected in the MOI 100 infection group. After 48 h, pronounced acidification was observed in both the MOI 50 and MOI 100 groups. Notably, Sia-GP treatment partially alleviated the infection-associated decrease in pH, whereas DeSia-GP and α2,3-DeSia-GP showed weaker effects. These results indicated that SAα2-3Gal attenuated *S. mutans*-induced acidification of the culture environment.

#### 2.6.2. Effects of Sia-GP on HOK Cell Clonogenic Capacity

The clonogenic capacity of HOK cells was further assessed by colony formation assay. As shown in Figure 6F, *S. mutans* infection markedly reduced the number of colonies formed by HOK cells. Sia-GP at 1.0 μg/μL partially alleviated the reduction in colony formation induced by *S. mutans* infection, while DeSia-GP and α2,3-DeSia-GP at the same concentration exhibited weaker effects.

#### 2.6.3. Effects of Sia-GP on HOK Cell Migration

Cell migration was evaluated using a wound-healing assay. As shown in Figure 6G, *S. mutans* infection significantly impaired wound closure in HOK cells. Treatment with Sia-GP (1.0 μg/μL) partially alleviated the impairment of HOK cell migration induced by *S. mutans* infection, whereas DeSia-GP and α2,3-DeSia-GP showed weaker effects.

### 2.7. Effects of SAα2-3Gal Glycan Structures on S. mutans-Induced Inflammatory Responses in HOK Cells

**Figure 7:**
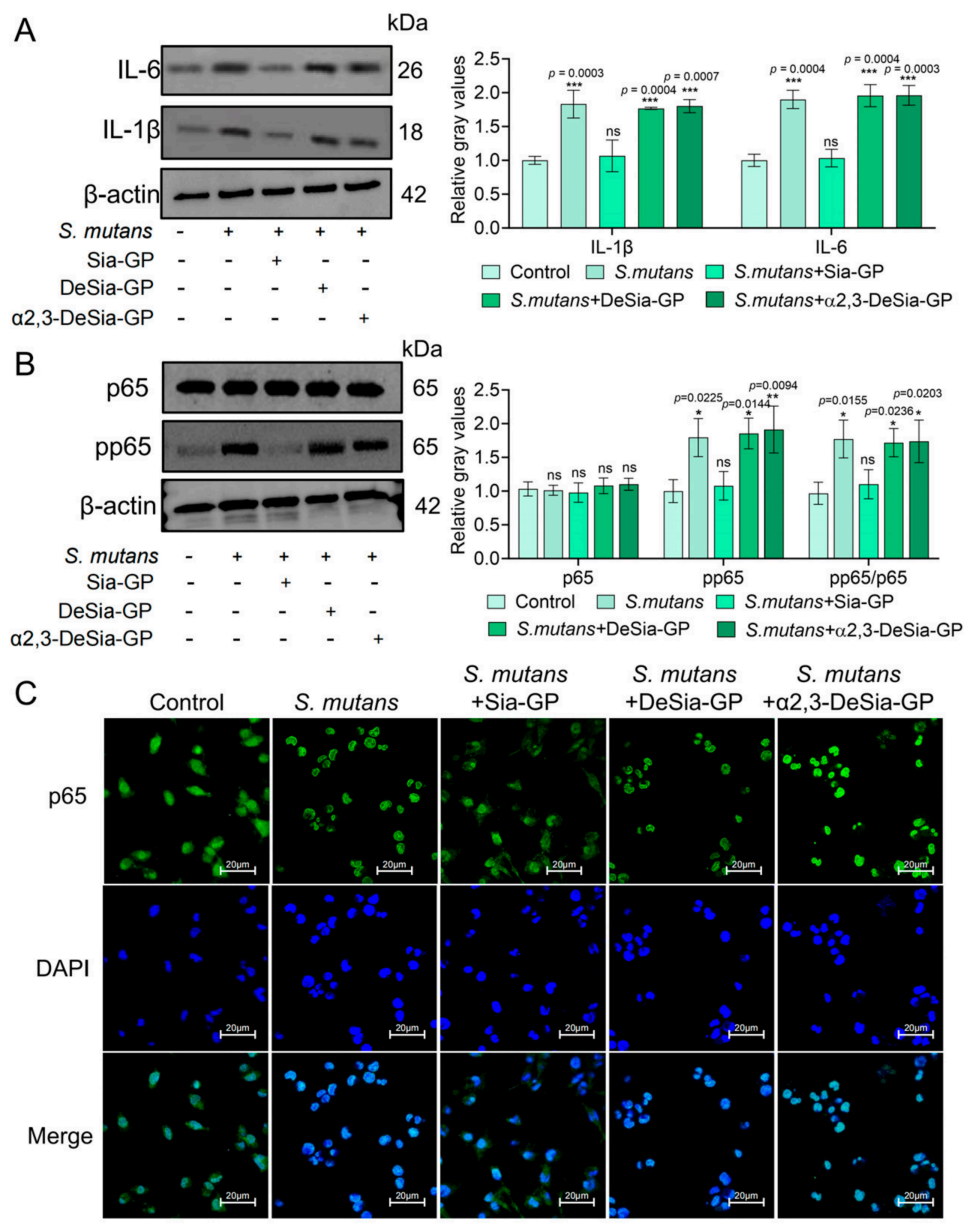
Effects of SAα2-3Gal glycan structures on *S. mutans*-induced inflammatory responses in HOK cells. (**A**) Western blot analysis showing the expression levels of pro-inflammatory cytokines IL-1β and IL-6 in HOK cells. *S. mutans* infection markedly increased cytokine expression, whereas treatment with Sia-GP attenuated this infection-induced increase in IL-1β and IL-6 levels. DeSia-GP and α2,3-DeSia-GP treatments showed diminished inhibitory effects. (**B**) Western blot analysis of total and phosphorylated p65. *S. mutans* infection increased p65 phosphorylation, which was effectively suppressed by Sia-GP treatment. DeSia-GP and α2,3-DeSia-GP exhibited minimal effects. (**C**) Representative confocal images of p65 nuclear translocation. *S. mutans* infection enhanced p65 accumulation in the nucleus, while Sia-GP treatment reduced infection-induced p65 nuclear translocation. DeSia-GP and α2,3-DeSia-GP treatments showed weaker effects. Nuclei were stained with DAPI (blue), and p65 was labeled with Alexa Fluor 488-conjugated goat anti-rabbit IgG (H + L) (green). Sia-GP—sialylated glycoprotein; DeSia-GP—desialylated glycoprotein; α2,3-DeSia-GP—α2,3-desialylated glycoprotein. Data are presented as mean ± SD from three independent experiments (*n* = 3), each performed in triplicate. ns—not significant; * *p* < 0.05, ** *p* < 0.01, *** *p* < 0.001.

Since *S. mutans* infection reduced HOK cell viability, proliferation, and migration, infection-associated inflammatory responses were further examined to characterize the cellular consequences of bacterial challenge. Western blot results showed that *S. mutans* increased the expression of IL-1β and IL-6 compared with the control group. Sia-GP treatment attenuated this infection-induced increase in inflammatory cytokine expression, whereas DeSia-GP and α2,3-DeSia-GP showed weaker effects (Figure 7A). In addition, *S. mutans* increased p65 phosphorylation and promoted its nuclear translocation. Sia-GP treatment reduced these infection-induced changes, while DeSia-GP and α2,3-DeSia-GP showed limited effects (Figure 7B,C). These results suggested that SAα2-3Gal structures attenuated inflammatory responses induced by *S. mutans* infection in HOK cells.

## 3. Discussion

Glycosylation is a ubiquitous and highly diverse biological modification that occurs across all domains of life and plays fundamental roles in numerous physiological processes. Glycans attached to proteins contribute to protein folding, molecular stability, cell communication, and immune regulation [2,7]. Beyond these intrinsic biological functions, glycans are increasingly recognized as critical mediators of host–microbe interactions, with specific glycan–lectin interactions influencing microbial adhesion, signaling, and immune responses [25]. In mucosal environments, glycoconjugates present on epithelial surfaces and in secreted fluids they provide carbohydrate ligands that can be recognized by microbial adhesins and lectins, thereby influencing bacterial adhesion, colonization, and community composition [11]. In addition to endogenous glycans associated with host tissues, exogenous glycans can also modulate microbial behavior by competing with host receptors or interfering with pathogen-associated processes. For instance, isolated mucin glycans have been shown to suppress virulence gene expression and biofilm formation in opportunistic pathogens such as *Pseudomonas aeruginosa* and to downregulate quorum sensing and competence in *S. mutans* [16,19,26]. These observations suggest that specific glycan motifs may function as modulators of microbial pathogenicity and contribute to the maintenance of microbial homeostasis.

*S. mutans* is a common opportunistic pathogen in the pediatric oral microbiota, whose ecological behavior is highly sensitive to host factors [27]. The pathogenicity of *S. mutans* is primarily mediated by a set of well-characterized virulence traits, including sucrose-dependent biofilm formation catalyzed by glucosyltransferases GtfB, GtfC, and GtfD, which produce EPS to consolidate the biofilm matrix, as well as acid production via sugar fermentation coupled with an efficient acid-tolerance response that enables survival under acidic conditions. These factors collectively enable *S. mutans* to establish a cariogenic biofilm, outcompete commensal species, and drive localized pH drops that promote enamel demineralization and subsequent caries development [28,29]. Thus, modulation of these virulence-associated processes is closely associated with the control of cariogenic microbial behaviors and the maintenance of oral microbial homeostasis. Previous studies have observed that children exhibit lower levels of SAα2-3Gal glycan structures in their oral cavity, which may influence *S. mutans* colonization and behavior [18]. To explore the biological significance of this glycan alteration, bovine milk-derived sialylated glycoproteins (Sia-GP) were employed as a glycan-defined source enriched in terminal sialylated structures. Although derived from bovine milk, the SAα2-3Gal glycans examined here are also present on human salivary glycoproteins, and the presence of SAα2-3Gal-linked glycans in Sia-GP has been confirmed by lectin microarray, lectin blotting, LC-MS/MS, and MALDI-TOF/TOF-MS analyses [30,31,32]. Therefore, bovine milk-derived glycoproteins were used here as an experimental surrogate for investigating the functional effects of SAα2-3Gal-containing structures rather than as physiological equivalents of human salivary glycoproteins.

In the present study, Sia-GP exerted only a minor and transient inhibitory effect on bacterial growth, primarily manifested as a slight delay in the early growth phase, while no significant differences were observed in the final biomass after 24 h. One possible explanation is that the effective concentration of intact Sia-GP gradually decreased during culture due to continuous bacterial utilization, adsorption, or structural modification, thereby weakening its inhibitory influence over time [33]. In contrast to its limited impact on proliferation, Sia-GP markedly reduced the acidogenic activity and EPS production of *S. mutans* and downregulated key glucosyltransferase genes (*gtfB*, *gtfC*, and *gtfD*) as well as acid-tolerance genes (*atpD* and *aguD*). This dissociation between growth maintenance and attenuation of virulence-associated phenotypes is consistent with observations reported for several anti-virulence agents. For example, luteolin and resveratrol have been shown to suppress biofilm formation, EPS synthesis, and acid production at concentrations that exert minimal effects on bacterial viability, suggesting that virulence-related physiological processes may be more sensitive to external modulation than overall bacterial proliferation [34,35]. Mechanistically, the observed dissociation may be attributed to the metabolic flexibility of *S. mutans*. Although glucosyltransferase-dependent extracellular glucan synthesis is a major contributor to biofilm matrix formation and cariogenic virulence, it represents one specific branch of sucrose utilization rather than the sole determinant of bacterial energy production and biomass accumulation. Under nutrient-rich culture conditions, *S. mutans* can utilize available carbohydrates and other nutrient sources through multiple metabolic routes to support cellular replication, even when resources allocated toward extracellular matrix production or acid-associated pathways are altered [36,37]. In addition, endpoint OD595 measurements primarily reflect the accumulated bacterial biomass at the end of incubation and do not directly represent the dynamic processes of substrate utilization, metabolic flux distribution, or acid production throughout cultivation. Thus, the comparable final biomass observed between control and Sia-GP-treated groups does not necessarily indicate identical metabolic states. Sia-GP may influence the allocation of metabolic resources between biomass maintenance and virulence-associated functions, resulting in reduced extracellular glucan production and acidogenic activity while preserving sufficient metabolic capacity for bacterial proliferation [38,39]. However, intracellular metabolic processes, including glycolytic flux, ATP generation, and carbon distribution, were not directly assessed in the present study. Therefore, the proposed metabolic interpretation remains a hypothesis, and further metabolomic and biochemical investigations will be required to determine whether Sia-GP induces broader metabolic reprogramming in *S. mutans*.

Among the observed virulence-associated alterations, the reduction in EPS production and glucosyltransferase-associated responses provides a potential entry point for further mechanistic exploration. Given that intact Sia-GP (~35 kDa) is unlikely to be efficiently internalized and that *S. mutans* lacks endogenous sialidase activity, the observed effects are more likely to involve extracellular or cell surface-associated processes rather than intracellular uptake of intact glycoproteins [40,41]. Unlike intracellular metabolic enzymes involved in acid production, glucosyltransferases are extracellular biofilm-associated proteins that function within the biofilm matrix, making them potential components involved in glycan-associated modulation of biofilm development [42]. To explore whether extracellular glucosyltransferases may participate in the biological effects associated with SAα2-3Gal structures, molecular docking and molecular dynamics simulations were performed. SAα2-3Gal showed favorable predicted interactions with GtfB, GtfC, and GtfD, and the predicted complexes remained relatively stable during molecular dynamics simulations, providing computational support for further investigation of whether extracellular glucosyltransferases participate in SAα2-3Gal-mediated effects. Based on the current findings, the reduction in biofilm formation and EPS production may involve multiple potential mechanisms. One possibility is that Sia-GP may influence extracellular biofilm-associated processes through interactions with or modulation of matrix-associated components, including glucosyltransferases, thereby affecting glucan-associated biofilm development [43]. Alternatively, and potentially in parallel, these effects may involve indirect regulation mediated by bacterial sensing systems and regulatory networks responding to extracellular environmental cues. *S. mutans* coordinates virulence-associated traits through multiple regulatory pathways. For example, the *VicRK* system regulates cell wall metabolism, biofilm formation, and gtf gene expression, whereas *LiaSR* contributes to responses associated with cell envelope stress. In addition, *CovRS* functions as a global transcriptional regulator of virulence-associated genes, *CcpA* links carbon metabolism with biofilm-related behaviors, and *ComDE* participates in quorum sensing-dependent adaptation and community development [44,45,46,47]. Therefore, extracellular exposure to Sia-GP may be sensed indirectly through bacterial surface-associated regulatory systems or changes in the extracellular microenvironment, which could subsequently influence gtf-associated transcriptional programs. However, no experimental evidence currently establishes a direct link between SAα2-3Gal-containing structures and these regulatory systems, and the involvement of these pathways remains a hypothesis requiring further investigation. Similarly, the predicted SAα2-3Gal–glucosyltransferase interactions are currently based on computational analyses and should be interpreted as structural hypotheses rather than direct evidence of functional recognition. Future studies combining glucosyltransferase activity assays, direct glycan–protein interaction measurements (such as SPR, ITC, or MST), and genetic approaches targeting relevant regulatory pathways will be necessary to determine whether SAα2-3Gal-mediated effects primarily involve extracellular biofilm-associated interactions, bacterial sensing systems, or a combination of these processes.

Beyond its effects on bacterial virulence-associated traits, Sia-GP treatment in the HOK infection model was accompanied by attenuation of several infection-associated host alterations, including reduced cell viability, clonogenicity, and migration, together with decreased IL-1β and IL-6 expression and reduced NF-κB activation. Because extracellular acidification represents one important route through which *S. mutans* can affect epithelial cell function [48], whether the observed host alterations were accompanied by changes in the co-culture pH was further examined. The results showed that *S. mutans* infection induced a time- and MOI-dependent decrease in extracellular pH, whereas Sia-GP partially attenuated this acidification. Extracellular acidification has been reported to promote inflammatory signaling and cellular stress responses through acid-sensitive signaling pathways, including acid-sensing ion channels and proton-responsive GPCRs [49,50,51]. Thus, reduced extracellular acidification may have contributed to the attenuated host responses observed in the present study. Nevertheless, extracellular acidification represents only one of multiple virulence-associated mechanisms of *S. mutans.* The attenuated host responses observed in the present study are more likely to reflect the combined consequence of reduced bacterial virulence rather than the effect of any single virulence-associated factor. However, the relative contribution of individual virulence-associated factors, including extracellular acidification, biofilm-associated changes, and other bacterial responses, remains unclear.

Among the diverse glycan structures involved in host–microbe interactions, sialylated glycans are of particular interest due to the exposed position of sialic acids on glycan chains, which allows them to engage microbial adhesins, lectins, and host immune receptors [7,52]. Sialic acids usually occupy the terminal positions of glycans and are predominantly attached to underlying galactose residues through α2-3 or α2-6 linkages. In mammals, α2-3 linkages are often enriched in milk and mucosal secretions, whereas α2-6 linkages are more prevalent on serum N-glycans [53,54]. The SAα2-3Gal motif is a prominent subtype in host–microbe interactions, serving as a preferential recognition target for microbial adhesins and lectins, and can act as decoy receptors to interfere with microbial adhesion or modulate innate immune responses [12,55]. In the oral cavity, terminal sialylated glycan structures are also present on major salivary mucins, such as MUC5B and MUC7. These highly glycosylated mucins contain diverse O-linked glycans, including sialylated structures, which contribute to interactions with oral microorganisms. For example, Neu5Acα2-3Gal-containing structures have been identified on human MUC7 and can participate in recognition by sialic acid-binding adhesins of oral streptococci, thereby influencing mucin–bacteria interactions [56,57]. Similarly to these salivary mucins, the biological activity of Sia-GP observed in the present study is likely associated not only with the protein backbone but also with the SAα2-3Gal-containing glycan structures it carries. Thus, the presence of conserved sialylated glycan motifs across different glycoprotein backbones suggests that specific glycan structures themselves play a role in host–microbe interactions.

The regulatory potential of glycans in shaping *S. mutans* virulence is further supported by studies on other host- and diet-derived carbohydrate structures, which can interfere with bacterial adhesion, biofilm formation, and metabolic activity, thereby limiting pathogenicity [58]. For example, oligosaccharides in human milk and certain plant-derived glycans have been shown to reduce biofilm formation and attenuate acid production in *S. mutans* [59,60], while other carbohydrate-based compounds can modulate bacterial gene expression and stress responses linked to virulence, resulting in compromised biofilm integrity and reduced pathogenic potential. Unlike the SAα2-3Gal motif examined in the present study, many of these glycans lack sialic acid residues and may function through distinct mechanisms, such as direct competition for sugar-binding sites on glucosyltransferases or chelation of calcium ions required for biofilm integrity [61,62]. Collectively, these findings indicate that structurally diverse glycans can influence *S. mutans* pathogenic behaviors through multiple mechanisms and highlight the potential of host glycan patterns as modulators of oral microbial ecology.

Several limitations of the present study should be acknowledged. First, although bovine milk-derived Sia-GP shares conserved terminal sialylated glycan motifs with human salivary glycoconjugates, differences remain in the protein backbone and overall glycosylation profile. Therefore, the present findings should be interpreted as reflecting the biological contribution of the SAα2-3Gal glycan motif rather than representing the full complexity of the human oral glycan environment. Second, although Sia-GP characterization was supported by linkage-specific neuraminidase digestion and MAL-II lectin blotting, additional orthogonal glycoanalytical approaches, such as SNA-based detection and comprehensive glycomic profiling, would further strengthen the structural assignment of individual sialylated glycan motifs. Finally, the present study employed a single-species *S. mutans* model to dissect the functional role of SAα2-3Gal-containing glycans. However, oral biofilms are complex multispecies ecosystems in which microbial interactions and glycan-active enzymes may influence the stability, accessibility, and biological effects of host-derived glycans. In particular, sialidase-producing oral microorganisms, such as certain *Streptococcus* and *Actinomyces* species, may modify terminal sialic acid structures, potentially affecting the persistence of SAα2-3Gal-containing glycans within mature biofilms [57]. Therefore, future studies using defined multispecies biofilm models will be essential to determine whether the regulatory effects observed here are maintained under physiologically relevant oral ecological conditions.

Collectively, these findings suggest that host glycan motifs play an important role in regulating bacterial behavior and highlight several directions for future research. A key priority is to clarify the molecular basis of SAα2-3Gal recognition by *S. mutans*, including the identification of specific bacterial surface proteins involved in this interaction. In parallel, it will be important to determine which structural features of the glycan are required for its biological activity, in order to better define structure–function relationships. From a translational standpoint, systematic evaluation of such glycan mimetics could pave the way for novel microbiome-modulating interventions in oral health, extending beyond caries prevention to other dysbiosis-related conditions.

## 4. Materials and Methods

### 4.1. Bacterial Culture and Cell Culture

*S. mutans* (CCTCC AB 99010) was obtained from the China Center for Type Culture Collection, and recovered in brain heart infusion (BHI) solid medium (Hopebio, Qingdao, China) at 37 °C for 24 h. Subsequently, the strain was cultured in BHI liquid medium (Hopebio, Qingdao, China) at 37 °C for 24 h. After activation, the strain was stored at −80 °C in 20% glycerin for longer preservation. The bacteria were grown until they reached the logarithmic growth phase before experiments. Human oral keratinocytes (HOKs), derived from normal oral mucosal epithelium, were used as an in vitro model to investigate host–microbe interactions in the oral cavity. HOKs were purchased from Otwo Biotech Inc. (Shenzhen, China; Cat No. HTX2966) and maintained in DMEM (HyClone, Waltham, MA, USA) supplemented with 10% fetal bovine serum, 100 U/mL penicillin, and 100 μg/mL streptomycin at 37 °C in a humidified atmosphere containing 5% CO_2_.

### 4.2. Preparation and Characterization of Glycoproteins

Sia-GP were enriched and purified from milk according to previously described methods [30,31,32]. DeSia-GP and α2,3-DeSia-GP were obtained by enzymatic removal of sialic acid residues from Sia-GP. Specifically, global desialylation (DeSia-GP) was performed using α2-3,6,8 Neuraminidase (New England Biolabs, Ipswich, MA, USA; Cat. No. P0720) in 1× GlycoBuffer (50 mM sodium acetate, 5 mM CaCl_2_, pH 5.5) at 37 °C for 1 h, followed by heat inactivation at 65 °C for 15 min. For linkage-specific desialylation (α2,3-DeSia-GP), an α2-3-specific neuraminidase (New England Biolabs, Ipswich, MA, USA; Cat. No. P0743) was applied under similar incubation conditions to selectively remove α2-3-linked sialic acid residues. α2,3-DeSia-GP was used to specifically assess the role of SAα2-3Gal glycan structures, whereas DeSia-GP served as a global sialic acid-removed control to confirm that the observed effects were not due to other sialic acid residues. The integrity and protein profiles of all glycoprotein preparations were evaluated by SDS-PAGE followed by silver staining. The efficiency and specificity of desialylation were further verified by lectin blotting using biotinylated MAL-II (Vector Laboratories, Newark, CA, USA; No. B-1265), which specifically recognizes α2-3-linked sialic acid residues. All glycoprotein preparations were dissolved in sterile buffer and stored at −20 °C until use.

### 4.3. Growth Curve Assay

The growth of *S. mutans* was monitored in the presence of different glycoprotein preparations. Sia-GP were added to bacterial cultures at final concentrations of 0.4, 0.8, and 1.0 μg/μL. The culture without Sia-GP served as the blank control. To investigate the role of the SAα2-3Gal glycan structures, DeSia-GP and α2,3-DeSia-GP were added at a concentration of 1.0 μg/μL as control treatments. The bacterial suspension was incubated at 37 °C, and the absorbance at 595 nm was measured every 2 h using a microplate reader (BioTek Instruments, Inc., Winooski, VT, USA) with Gen5 software (version 3.04) to plot the growth curve.

### 4.4. Acidification and Glycolytic Activity Assays

The acidogenic ability of *S. mutans* was evaluated by measuring the pH of the culture supernatant. The same experimental groups as described in Section 4.3 were used. Briefly, *S. mutans* was inoculated into BHI broth supplemented with 1% (*w*/*v*) sucrose in the presence or absence of different concentrations of Sia-GP. The mixtures were incubated at 37 °C, and samples were collected at 0, 3, 6, 12, and 24 h. At each time point, the cultures were centrifuged to obtain the supernatant, and the pH values were determined using a pH meter (PHS-3E, Shanghai INESA Scientific Instrument Co., Ltd., Shanghai, China), which was calibrated prior to each measurement using standard buffer solutions at pH 4.00, 7.00, and 10.00 according to the manufacturer’s instructions.

For glycolytic activity assessment, bacterial cells were harvested from mid-log phase cultures and washed twice with 50 mM KCl and 1 mM MgCl_2_ buffer (pH 7.0) to remove residual medium. Glycolysis was initiated by adding glucose (final concentration 1%, *w*/*v*), and the pH decrease in the suspension was monitored over time using the same calibrated pH meter. The rate of pH change was used to reflect glycolytic activity.

### 4.5. Crystal Violet Biofilm Assay

Biofilm formation of *S. mutans* was evaluated using a crystal violet staining assay. *S. mutans* was inoculated into BHI broth supplemented with 1% (*w*/*v*) sucrose in the presence or absence of different glycoprotein treatments. After incubation at 37 °C for 24 h, the culture medium was removed and the wells were washed three times with PBS to remove planktonic cells. The attached biofilms were stained with 0.1% crystal violet (Beyotime Biotechnology, Shanghai, China) for 15 min and washed with distilled water. The bound dye was dissolved in 95% ethanol, and the absorbance was measured at 595 nm using a microplate reader (BioTek Instruments, Inc., Winooski, VT, USA) with Gen5 software (version 3.04).

### 4.6. Confocal Laser Scanning Microscopy Analysis of Biofilm Structure

Confocal laser scanning microscopy (CLSM) analysis of biofilm structure was performed as previously described [43]. *S. mutans* biofilms were cultivated for 24 h on flat abiotic surfaces under the same experimental conditions. Sia-GP, DeSia-GP, or α2,3-DeSia-GP were added at indicated concentrations. After incubation, the biofilms were gently washed twice with PBS to remove non-adherent cells. The samples were stained with SYTO 9 (5 μM in PBS) for 20 min in the dark at room temperature. Excess dye was removed by rinsing with PBS. Images were acquired using a Leica confocal laser scanning microscope (Leica TCS SP8, Leica Microsystems, Wetzlar, Germany). SYTO 9 was excited at 488 nm and emission was collected at 500–550 nm. Z-stack images were acquired at 1 μm intervals using a 20× objective. Three-dimensional reconstruction and quantitative analysis of biofilm thickness and surface coverage were performed using the Leica LAS X software (version 3.5.7).

### 4.7. Scanning Electron Microscopy

Scanning electron microscopy (SEM) was performed to evaluate the morphological characteristics of *S. mutans* biofilms. Biofilms were cultivated for 24 h under identical experimental conditions using two complementary models: a flat abiotic surface and hydroxyapatite (HA) discs. The flat abiotic surface was used as a standardized substrate to ensure reproducible biofilm formation and facilitate comparison across experimental groups. In contrast, HA discs were employed to mimic enamel-like tooth surfaces, providing a biologically relevant substrate for assessing bacterial adhesion and biofilm architecture under oral-like conditions. For HA-based biofilm formation, HA discs were pre-coated with artificial saliva overnight at 37 °C under gentle agitation to establish an acquired salivary pellicle, as previously described [63], and then transferred into culture wells for bacterial inoculation. After incubation, samples were gently washed twice with PBS to remove non-adherent bacteria, fixed with 2.5% glutaraldehyde for 1 h at room temperature, dehydrated through a graded ethanol series (35%, 50%, 75%, 90%, and 100%; 15 min each), sputter-coated with gold, and observed using SEM (SU8600, Hitachi High-Technologies Corporation, Tokyo, Japan) to assess biofilm adhesion and structural organization.

### 4.8. Extracellular Polysaccharide Quantification

The production of EPS by *S. mutans* was quantified by measuring the amounts of WSG and WIG using the anthrone–sulfuric acid method. *S. mutans* was cultured in BHI broth supplemented with 1% (*w*/*v*) sucrose with indicated glycoprotein treatments for 24 h. Subsequently, the culture supernatant was removed and the biofilm formed at the bottom of the plate was carefully scraped and transferred into centrifuge tubes. The samples were centrifuged at 4000 rpm for 10 min. The pellets were washed twice with PBS and centrifuged again. The collected supernatants were combined and used as the WSG fraction. The remaining pellets were resuspended in 0.4 mol/L NaOH and incubated at 37 °C for 2 h to extract water-insoluble glucans. The suspension was then centrifuged at 6000 rpm for 10 min at 4 °C, and the resulting supernatant was collected as the WIG fraction. For polysaccharide quantification, the samples were mixed with anthrone–sulfuric acid reagent at a volume ratio of 1:3 and heated at 95 °C for 10 min. After cooling to room temperature, the reaction mixtures were transferred to a 96-well microplate, and the absorbance was measured at 630 nm using a microplate reader (BioTek Instruments, Inc., Winooski, VT, USA) with Gen5 software (version 3.04).

### 4.9. Quantitative Real-Time PCR (qRT-PCR) Analysis

*S. mutans* was cultured in BHI broth supplemented with 1% (*w*/*v*) sucrose in the presence of indicated glycoprotein treatments for 24 h. Cells were then collected, resuspended in PBS, and centrifuged at 4000× *g* for 10 min. The supernatant was discarded, and total RNA was extracted using a bacterial RNA isolation kit (Sangon Biotech, Shanghai, China) according to the manufacturer’s instructions. The extracted RNA was reverse-transcribed into cDNA using the PrimeScript RT Reagent Kit (TaKaRa, Kusatsu, Japan). Quantitative real-time PCR (qRT-PCR) was performed using TB Green Fast qPCR Mix (TaKaRa, Japan) on an ABI ViiA™ 7 real-time PCR system (Applied Biosystems, Foster City, CA, USA). Gene-specific primers targeting glucosyltransferase-related genes (*gtfB*, *gtfC*, and *gtfD*) and acid-associated genes (*atpD* and *aguD*) were used, with 16S rRNA as the internal reference gene (Table A3). Relative expression levels of target genes were calculated using the 2*^−ΔΔCt^* method.

### 4.10. Molecular Docking and Molecular Dynamics Simulations

The three-dimensional structures of GtfB, GtfC, and GtfD from *Streptococcus mutans* were obtained from UniProt. Water molecules, ligands, and non-essential ions were removed from the protein structures, and polar hydrogen atoms were added using AutoDock Tools (version 1.5.7). Twelve representative mono- and oligosaccharide structures were built using Chem3D and optimized with the MM2 force field. Molecular docking simulations were performed using AutoDock Vina (version 1.2.0) to evaluate the binding preferences of the 12 glyco-ligands with GtfB, GtfC, and GtfD. The docking grid box was centered on the catalytic or substrate-binding regions of each protein and sized to fully encompass the entire binding pocket. Default parameters were applied, and the conformations with the lowest binding energies were selected for subsequent analysis. All docking results were visualized using PyMOL (version 2.5.0) to analyze hydrogen bonding, hydrophobic interactions, and other potential carbohydrate–protein contacts. To evaluate the reliability and reproducibility of the docking protocol, SAα2-3Gal and SAα2-6Gal were subjected to re-docking simulations against the binding sites of GtfB, GtfC, and GtfD. Thirty independent docking runs were performed for each ligand–protein pair, and the root-mean-square deviation (RMSD) between the top-ranked poses was calculated. An RMSD value below 2.0 Å was considered indicative of acceptable docking reproducibility.

To further investigate the dynamic stability of the predicted glycan–protein complexes, molecular dynamics (MD) simulations were performed using GROMACS 2022.3. Glycan parameters were generated using AmberTools22 with the GAFF force field, and RESP charges were assigned to the glycan molecules. The protein–glycan complexes were simulated using the Amber99SB-ILDN force field with the TIP3P water model. After energy minimization and equilibration under NVT and NPT conditions, 100 ns production simulations were carried out at 300 K and 1 bar. The stability of the complexes was evaluated by root-mean-square deviation (RMSD) analysis, and the binding free energies were estimated using the MM/GBSA method.

### 4.11. Cytotoxicity Assay on HOKs

HOK cells were seeded in 96-well plates at a density of approximately 3000 cells per well. For all co-culture experiments, *S. mutans* was first cultured to the logarithmic phase in BHI broth supplemented with 1% (*w*/*v*) sucrose, harvested by centrifugation, washed with sterile PBS, and subsequently resuspended in sucrose-free DMEM at the indicated multiplicity of infection (MOI) before being co-cultured with HOK cells. Sia-GP, DeSia-GP, or α2,3-DeSia-GP were added at indicated concentrations. Cell viability was assessed using a Cell Counting Kit-8 (CCK-8, Beyotime Biotechnology, Shanghai, China) according to the manufacturer’s instructions at 0, 12, 24, 36 and 48 h after treatment. To evaluate infection-associated acidification during co-culture, culture supernatants were collected at 24 h and 48 h under the same experimental conditions used for the cytotoxicity assay. Briefly, 150 μL of supernatant was collected from each well and the pH was immediately measured using the same calibrated pH meter as described in Section 4.4.

### 4.12. Colony Formation Assay

HOK cells were seeded in 6-well plates at 500 cells per well. Bacterial suspensions were prepared as described in Section 4.11. Cells were cultured under different treatments for 10–14 days, with medium changes every 3 days. Colonies were fixed with 4% paraformaldehyde (Servicebio, Wuhan, China), stained with 0.5% crystal violet (Beyotime Biotechnology, Shanghai, China), and colonies containing more than 50 cells were counted.

### 4.13. Wound-Healing Assay

HOK cells were grown to ~90% confluence in 6-well plates. A uniform scratch was made, detached cells were removed with PBS, and fresh medium containing the indicated glycoprotein treatments and bacterial suspensions (prepared as described in Section 4.11) was added. Images were captured at 0 and 12 h using an inverted microscope (Optika Microscopes, Ponteranica, Italy), and wound closure was quantified with ImageJ (version 1.54).

### 4.14. Western Blotting

Proteins were extracted from HOK cells co-cultured with *S. mutans* using RIPA buffer (Applygen, Beijing, China). Thirty micrograms of protein per lane was separated on 10% SDS-PAGE gel and transferred to 0.45 μm PVDF membranes (Millipore, Billerica, MA, USA). Membranes were blocked with 5% non-fat milk at room temperature for 1 h and then incubated overnight at 4 °C with the following primary antibodies: IL-1β (Proteintech, Wuhan, China), IL-6 (Proteintech, Wuhan, China), P65 (Abways, Shanghai, China), p-P65 (Abways, Shanghai, China), and β-actin (Abways, Shanghai, China). After washing, membranes were incubated with secondary antibodies at 25 °C for 1 h, and protein bands were visualized using an enhanced chemiluminescence system (ECL kit, Yeasen Biotechnology, Shanghai, China). Band intensities were quantified by densitometry using ImageJ software (version 1.54). IL-1β and IL-6 signals were normalized to β-actin, while p-p65 signals were normalized to total p65.

### 4.15. Nuclear Translocation Assay

HOK cells were seeded in confocal dishes and subjected to different treatments with sialylated glycoproteins and their modified forms, followed by co-culture with *S. mutans*. After treatment, cells were washed with PBS, fixed with 4% paraformaldehyde for 15 min, and permeabilized with 0.1% Triton X-100 for 10 min at room temperature. Cells were blocked with 5% bovine serum albumin (BSA) in PBS for 1 h and incubated overnight at 4 °C with primary antibody against P65 (Abways, Shanghai, China). After washing, cells were incubated with Alexa Fluor 488-conjugated goat anti-rabbit IgG (H + L) (ABclonal, Wuhan, China) for 1 h at room temperature in the dark. Nuclei were stained with DAPI (Beyotime Biotechnology, Shanghai, China) for 5 min. Images were acquired using a Leica confocal laser scanning microscope (Leica TCS SP8, Leica Microsystems, Wetzlar, Germany).

### 4.16. Statistical Analysis

All experiments were performed using three independent biological replicates unless otherwise stated. Technical replicates within each experiment were averaged before statistical analysis. Data are presented as the mean ± standard deviation (SD). Representative images shown in figures are from one of three independent experiments. Statistical analysis was performed using GraphPad Prism (version10.0.2). Prior to performing ANOVA, the assumptions of parametric tests were evaluated by assessing the normality of model residuals and homogeneity of variance. Considering the limited number of biological replicates, residual distributions were also examined using Normal Q-Q plots. For time-course experiments, two-way ANOVA with repeated measures (treatment × time) followed by Dunnett’s multiple comparisons test was applied, with the control group as the reference. For all other multi-group comparisons, one-way ANOVA followed by Dunnett’s multiple comparisons test was used. For molecular docking data, statistical comparisons between paired groups were performed using the Wilcoxon matched-pairs signed-rank test. A value of *p* < 0.05 was considered statistically significant.

## 5. Conclusions

The present study demonstrated that SAα2-3Gal-containing glycoproteins attenuated multiple virulence-associated traits of *S. mutans*, including acidogenic activity, extracellular polysaccharide production, biofilm formation, and the expression of virulence-related genes. These changes were accompanied by reduced inflammatory responses and improved cellular status in *S. mutans*-infected HOK cells. Collectively, the findings support a role for SAα2-3Gal glycan structures in modulating bacterial behavior and host–microbe interactions and provide new insight into how alterations in host glycosylation patterns may influence susceptibility to cariogenic bacterial colonization.

## Data Availability

The original contributions presented in this study are included in the article. Further inquiries can be directed to the corresponding authors.

## Appendix A

**Table A1:** Effects of SAα2-3Gal on water-insoluble and water-soluble glucan production in *S. mutans*.

Group	Concent		Ratio
	WIG (μg/mL)	WSG (μg/mL)	(WIG/WSG)
Control	98.66 ± 7.46	64.81 ± 3.53	1.52
0.4 μg/μL Sia-GP	87.28 ± 7.43	62.11 ± 8.01	1.41
0.8 μg/μL Sia-GP	67.33 ± 5.76	47.21 ± 4.73	1.43
1.0 μg/μL Sia-GP	53.49 ± 3.70	38.17 ± 1.47	1.40
1.0 μg/μL DeSia-GP	92.91 ± 0.89	58.77 ± 7.96	1.58
1.0 μg/μL α2,3-DeSia-GP	92.44 ± 12.37	65.04 ± 2.20	1.42

WIG: water-insoluble glucan; WSG: water-soluble glucan (WSG).

**Table A2:** Docking energies of SAα2-3Gal and other carbohydrate ligands with *S. mutans* glucosyltransferases.

Carbohydrate Ligand	Binding Energies (kcal/mol)		
	GtfB	GtfC	GtfD
SAα2-3Gal	−8.0	−7.4	−7.1
SAα2-6Gal	−6.1	−5.4	−6.5
Fucα1-6GlcNAc	−6	−5.9	−5.8
Fucα1-2Gal	−5.6	−5.7	−6.3
Fucα1-3GlcNAc	−5.2	−5.1	−6.8
GlcNAc	−5.5	−4.7	−5.6
Gal	−5.8	−5.4	−6.2
GalNAc	−5.5	−4.7	−5.4
Glc	−5	−4.2	−5.4
Man	−5	−4.5	−5.2
Sia	−5.3	−5.3	−6.1
Xyl	−4.4	−4.2	−4.8

**Table A3:** Quantitative real-time PCR primers used in this study.

Primers	Sequences (5′–3′)
*gtfB*-F	TCTTCATTGACCAAGGAAATCGG
*gtfB*-R	TCCGGGGTGCATTATCTCTAC
*gtfC*-F	ATGATGGCTTATTACAGTGGCAA
*gtfC*-R	GTCGGAGATTCGTAGCTGGA
*gtfD*-F	GGTACATCCTCGACGGCATCT
*gtfD*-R	GTGCCTCTTGCTGCTTTCAC
*atpD-F*	CGTGCTCTCTCGCCTGAAATAG
*atpD-R*	ACTCACGATAACGCTGCAAGAC
*aguD-F*	ATCCCGTGAGTGATAGTATTTG
*aguD-R*	CAAGCCACCAACAAGTAAGG
16s RNA-F	GAGACACGGCCCAGACTCCT
16s RNA-R	CACCCATCGCTTGTCCTAATCTATG

**Table A4:** Binding free energy decomposition of SAα2-3Gal–GtfB/GtfC/GtfD complexes.

Contribution Components	GTFB-SAa2-3gal	GTFC-SAa2-3gal	GTFD-SAa2-3gal
ΔE_vdW_	−27.03 ± 0.84	−33.01 ± 1.50	−27.36 ± 0.42
ΔE_elec_	−84.54 ± 3.05	−65.25 ± 0.91	−89.87 ± 5.66
ΔE_GB_	87.95 ± 0.76	81.34 ± 0.23	98.21 ± 0.58
ΔE_surf_	−6.76 ± 0.04	−6.11 ± 0.01	−5.39 ± 0.03
ΔG_gas_	−111.57 ± 3.17	−98.26 ± 1.76	−117.23 ± 5.68
ΔG_solvation_	81.19 ± 0.76	75.23 ± 0.23	92.82 ± 0.58
ΔG_bind_	−30.38 ± 2.25	−23.03 ± 1.77	−24.41 ± 5.71

ΔE_vdW_—van der Waals energy; ΔE_elec_—electrostatic energy; ΔE_GB_—polar solvation energy; ΔE_surf_—nonpolar solvation energy; ΔG_gas_—gas-phase free energy; ΔG_solvation_—solvation free energy; ΔG_bind_—total binding free energy.
